# Multilevel approaches to address disparities in lung cancer screening: a study protocol

**DOI:** 10.1186/s43058-024-00553-4

**Published:** 2024-02-16

**Authors:** Randi M. Williams, Julia Whealan, Kathryn L. Taylor, Lucile Adams-Campbell, Kristen E. Miller, Kristie Foley, George Luta, Heather Brandt, Katharine Glassmeyer, Anu Sangraula, Peyton Yee, Kaylin Camidge, Joseph Blumenthal, Saumil Modi, Heather Kratz

**Affiliations:** 1 Lombardi Comprehensive Cancer Center, Cancer Prevention and Control Program, Georgetown University Medical Center, Washington, DC USA; 2 MedStar Health Research Institute, Washington, DC USA; 3 Wake Forest University School of Medicine, Winston-Salem, NC USA; 4 Department of Biostatistics, Bioinformatics, and Biomathematics, Georgetown University Medical Center, Washington, DC USA; 5 Epidemiology and Cancer Control Department, St. Jude Children’s Research Hospital, Memphis, TN USA; 6 MedStar Health, Columbia, MD USA; 7 The Catholic University of America, Washington, DC USA

**Keywords:** Lung cancer screening, Primary care, Disparities in lung cancer screening, Multilevel trials

## Abstract

**Background:**

Low-dose computed tomography (lung cancer screening) can reduce lung cancer-specific mortality by 20–24%. Based on this evidence, the United States Preventive Services Task Force recommends annual lung cancer screening for asymptomatic high-risk individuals. Despite this recommendation, utilization is low (3–20%). Lung cancer screening may be particularly beneficial for African American patients because they are more likely to have advanced disease, lower survival, and lower screening rates compared to White individuals. Evidence points to multilevel approaches that simultaneously address multiple determinants to increase screening rates and decrease lung cancer burden in minoritized populations. This study will test the effects of provider- and patient-level strategies for promoting equitable lung cancer screening utilization.

**Methods:**

Guided by the Health Disparities Research Framework and the Practical, Robust Implementation and Sustainability Model, we will conduct a quasi-experimental study with four primary care clinics within a large health system (MedStar Health). Individuals eligible for lung cancer screening, defined as 50–80 years old, ≥ 20 pack-years, currently smoking, or quit < 15 years, no history of lung cancer, who have an appointment scheduled with their provider, and who are non-adherent to screening will be identified via the EHR, contacted, and enrolled (*N* = 184 for implementation clinics, *N* = 184 for comparison clinics; total *N* = 368). Provider participants will include those practicing at the partner clinics (*N* = 26). To increase provider-prompted discussions about lung screening, an electronic health record (EHR) clinician reminder will be sent to providers prior to scheduled visits with the screening-eligible participants. To increase patient-level knowledge and patient activation about screening, an inreach specialist will conduct a pre-visit phone-based educational session with participants. Patient participants will be assessed at baseline and 1-week post-visit to measure provider-patient discussion, screening intentions, and knowledge. Screening referrals and screening completion rates will be assessed via the EHR at 6 months. We will use mixed methods and multilevel assessments of patients and providers to evaluate the implementation outcomes (adoption, feasibility, acceptability, and fidelity).

**Discussion:**

The study will inform future work designed to measure the independent and overlapping contributions of the multilevel implementation strategies to advance equity in lung screening rates.

**Trial registration:**

ClinicalTrials.gov, NCT04675476. Registered December 19, 2020.

Contributions to the literature

- Low-dose computed tomography (lung cancer screening) can reduce lung cancer-specific mortality by 20–24%, But utilization is low (3–20%)
- Lung cancer screening may be particularly beneficial for African American patients because they are more likely to have advanced disease, lower survival, and lower screening rates compared to White individuals.
- This study will test the effects of provider- and patient-level strategies for promoting equitable lung cancer screening utilization.
- This study is guided by the NIH Health Disparities Framework and uses mixed methods to evaluate the impact of multilevel implementation strategies on lung screening rates between African American and White patients.

## Background

The United States (US) Preventive Services Task Force (USPSTF) recommends annual lung cancer screening for asymptomatic individuals aged 50 to 80 years who have a ≥ 20 pack-year smoking history and currently smoke or have quit within the past 15 years (Grade B) [1]. Based on a systematic review of the evidence, including two large randomized controlled trials, the USPSTF concluded that screening high-risk individuals via low-dose computed tomography (CT) can reduce lung cancer mortality and may reduce all-cause mortality [1]. These trials included the National Lung Screening Trial (NLST), which reported that compared to chest X-ray, annual low-dose CT resulted in a 20% lung cancer-specific mortality reduction among individuals at high risk for lung cancer [2], and the Dutch NELSON trial, which demonstrated a 24% lung cancer mortality reduction among men who were screened versus not screened [3]. Despite the initial USPSTF recommendation released in 2013, the available estimates indicate screening uptake remains low, ranging from 3% in 2015 to 6.5–20% in 2019–2020 [4–6]. While the expanded criteria in 2021 increased the number of lung cancer screening-eligible individuals from 8 million to 14.5 million [7], barriers may still persist in the uptake of lung cancer screening [8].

Racial disparities in lung cancer predominantly affect Black or African American individuals (African Americans) in the US. Although African Americans smoke fewer cigarettes per day than White individuals (Whites), there are persistent differences in lung cancer rates between these groups [9]. African American men have the highest lung cancer incidence and death rates compared to all racial and ethnic groups. Relative to their White counterparts, African American women are at a lower risk of getting the disease, but have comparable cancer death rates [9]. Additionally, African Americans are diagnosed with lung cancer at an earlier age compared to Whites [10].

There are several drivers of lung cancer health disparities, including forms of oppression that span across the cancer continuum and occur at multiple levels (e.g., individual, societal) [11]. This includes differences in exposure to risk factors (e.g., greater exposure to air pollution and tobacco smoke), lack of insurance and barriers to healthcare access, historical medical racism and medical mistrust, discrimination within the healthcare system including differential discussion based on a patient’s race, and underrepresentation in clinical trials that inform the development of screening guidelines and novel therapies. It is also well documented that the tobacco industry has used long-standing predatory practices to target minoritized groups resulting in greater tobacco exposure and tobacco-related disparities, including lung cancer [12].

Lung cancer screening may be particularly beneficial for African Americans because they are more likely to have advanced disease and lower survival compared to Whites [13]. Additionally, data from the NLST suggested that lung cancer screening reduces lung cancer mortality to a greater extent in African Americans compared to all racial groups (hazard ratio, 0.61 vs. 0.86) [14]. However, lung cancer screening rates are consistently low across all racial and ethnic groups [6], and data suggest rates of uptake are lower among African Americans compared to Whites [15–17]. In the context of lung cancer screening, there are several factors that may contribute to racial disparities including (1) current screening guidelines do not take into account additional risk factors and differences in smoking patterns among individuals who may benefit from early detection, (2) socially disadvantaged areas that contribute to a higher smoking rate and higher lung cancer incidence, but have fewer accredited screening facilities creating an access issue, and (3) medical mistrust and the smoking-related stigma that can stand in the way of seeking screening or other forms of medical care [18]. Evidence points to the need for multilevel methods that simultaneously address multiple determinants to increase screening rates and decrease lung cancer morbidity and mortality in minoritized populations [19–21].

The National Institute on Minority Health and Health Disparities (NIMHD) Health Disparities Research Framework emphasizes the importance of using multilevel approaches to address disparities [22]. The framework identifies multiple health determinants, including behavior (e.g., preventive health behaviors, tobacco use exposure) and healthcare systems (e.g., systemic bias, patient-provider communication) that result in racial disparities in health outcomes. Within the lung cancer screening context, there are determinants at the *healthcare system-level* (e.g., lack of investment by health systems to identify lung cancer screening eligible patients); at the *provider-level* (e.g., provider time constraints, lack of familiarity with screening criteria, and racial bias); and at the *patient-level* (e.g., low awareness and knowledge about lung cancer screening, smoking-related stigma, and medical mistrust) [15, 18, 23]. There is a growing body of literature of multilevel interventions and implementation strategies that show promise in improving health outcomes and advancing health equity [21]. However, to date, there have only been four multilevel studies conducted on lung screening [24–27]. In a multilevel intervention that used best practice alerts and trainings for providers as well as marketing and patient navigation with language interpreters, they increased the number of lung cancer screenings compared to baseline with the highest rates among Hispanic patients, followed by Black patients, and non-Hispanic White patients [24]. Other studies have used system- and provider-level implementation strategies including automated reports and utilizing nurse practitioners to schedule and enroll patients into the screening program [25–27]. Colamonici and colleagues found an immediate and sustained increase in weekly lung cancer screening referrals by targeting providers and patients, but these studies noted barriers to successful implementation including implementation characteristics (e.g., organizational readiness, patient needs, and resources) and the need for future multisite projects [25–27].

Inreach is the process of identifying potentially eligible individuals within a clinic or health system via the EHR and attempting to connect them with evidence-based care [28]. Inreach, typically conducted by a screening program navigator or coordinator, is an approach that has been used widely in clinical settings and found to be effective for other screening types, such as colorectal, breast, and cervical cancers [29–32]. While there is currently a lack of research focusing on the utility of inreach for lung cancer screening, two studies used methods for identifying eligible patients in order to provide educational classes and interactive screening decision aids. They found these strategies can increase lung cancer screening-related knowledge and behaviors [32, 33]. In order to target key barriers to lung cancer screening at the patient-level, this study will utilize inreach to identify potentially eligible individuals via the EHR ahead of an upcoming clinic visit. Trained health education specialists will educate patients about lung cancer screening and promote patient activation for the individual to discuss screening at their next visit. The concept of patient activation has been used widely and it empowers patients to obtain the skills, knowledge, and motivation to participate as a member of the care team [34, 35]. Thus, the patient component will target lung screening knowledge and awareness along with other barriers (e.g., smoking-related stigma, medical mistrust) to encourage patient-provider discussion.

Clinician reminders are one of the Expert Recommendations for Implementing Change (ERIC) discrete implementation strategies that prompt clinicians to recall information or use a clinical innovation, like evidence-based screening recommendations. In general practice, provider prompts and best practice alerts have been effective in promoting evidence-based and guideline-concordant care. However, providers have reported “alert fatigue” suggesting that reminders are ignored [36–38]. To date, three studies have been conducted to evaluate the impact of clinician reminders on lung screening referrals and have found these to be effective approaches to improving lung cancer screening in primary care [24, 39, 40]. A pre-visit planning message is a type of clinician reminder that aims to help the provider conduct the clinic visit more effectively by gathering information ahead of time so they can devote more attention during the visit to discussing that information and responding to the patient’s questions. An advantage of this type of reminder is that it is not disruptive to the provider’s workflow like pop-up alerts may be. This type of clinician reminder may be especially important for lung cancer screening, because to be eligible for coverage, the Centers for Medicare & Medicaid Services guidelines require a shared decision-making visit [41]. This visit is required to be conducted by a healthcare provider with the patient prior to the first screening referral to discuss the benefits, risks, and limitations of screening, and smoking cessation. To address provider time constraints about lung screening, this study will send a pre-visit planning message as a reminder to discuss screening with eligible patients.

**Fig. 1:**
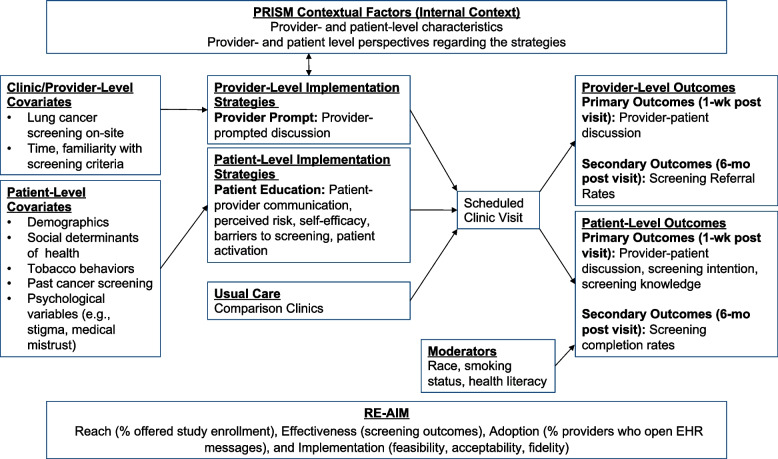
Conceptual framework

This study is guided by the Practical, Robust, Implementation & Sustainability (PRISM) Model, which includes the Reach, Effectiveness, Adoption, Implementation, Maintenance (RE-AIM) Framework [42, 43]. The planned trial will assess the effectiveness and feasibility of the multilevel implementation strategies to improve lung cancer screening utilization and to address racial disparities (Fig. 1). PRISM expands upon the RE-AIM framework and contains two parts: the contextual factors at individual and organizational levels and the RE-AIM outcomes. In this study, we will use RE-AIM to quantify the reach as measured by percent of providers and percent of patients offered study enrollment, effectiveness (screening outcomes), adoption (% providers who open EHR messages), and implementation (feasibility, acceptability, fidelity). This study will use mixed methods to conduct semi-structured interviews before (pre-) and after (post-) trial with MedStar leadership, referring providers, clinic staff, and participants.

## Methods/design

### Study aims

This study will target provider- and patient-level barriers in order to improve utilization of lung screening and to achieve equity in screening rates by conducting a quasi-experimental study comparing a Multilevel Intervention to Usual Care. The Multilevel Intervention will perform inreach to educate patients about screening prior to their visit. At the same time, an EHR communication tool, similar to email, will send a reminder to primary care providers prior to scheduled visits with screening-eligible patients.

The *specific aims* of the trial are to (1) test the impact of the multilevel strategies on primary outcomes (provider-patient discussion, screening intentions, and knowledge) and secondary outcomes (screening referrals and screening completion rates) and (2) evaluate the implementation outcomes (adoption, feasibility, acceptability, fidelity).

The *hypotheses* are (1) participants in the Multilevel Clinics who receive the provider- and patient-level strategies will be significantly more likely to report having a screening discussion, intending to be screened, and having greater knowledge compared to the Comparison Clinic participants; (2) the multilevel, bundled strategies will increase screening referrals made by providers in the Multilevel Clinics; and (3) completion rates among the participants will be higher among the Multilevel Clinic participants compared to the Comparison Clinic participants. Finally, we will explore whether Health Disparities Framework factors (e.g., race, education, health literacy) moderate the outcomes (e.g., we expect that African Americans will have significantly greater knowledge in the Multilevel Clinics vs. the Comparison Clinics, whereas the strategies will have less of an impact among Whites).

### Clinical setting and healthcare provider participants

MedStar Health, the largest not-for-profit health system in the Mid-Atlantic, includes more than 300 care locations including more than 60 ambulatory care centers, and primary and specialty care in academic medical centers and community-based clinics, 10 hospitals, and 33 urgent care clinics located in Baltimore, MD, Washington, DC, and Northern VA. This health system provides care to a diverse community which includes a large Black or African American population (44–73% in the participating clinics), and uninsured or underinsured individuals.

**Fig. 2:**
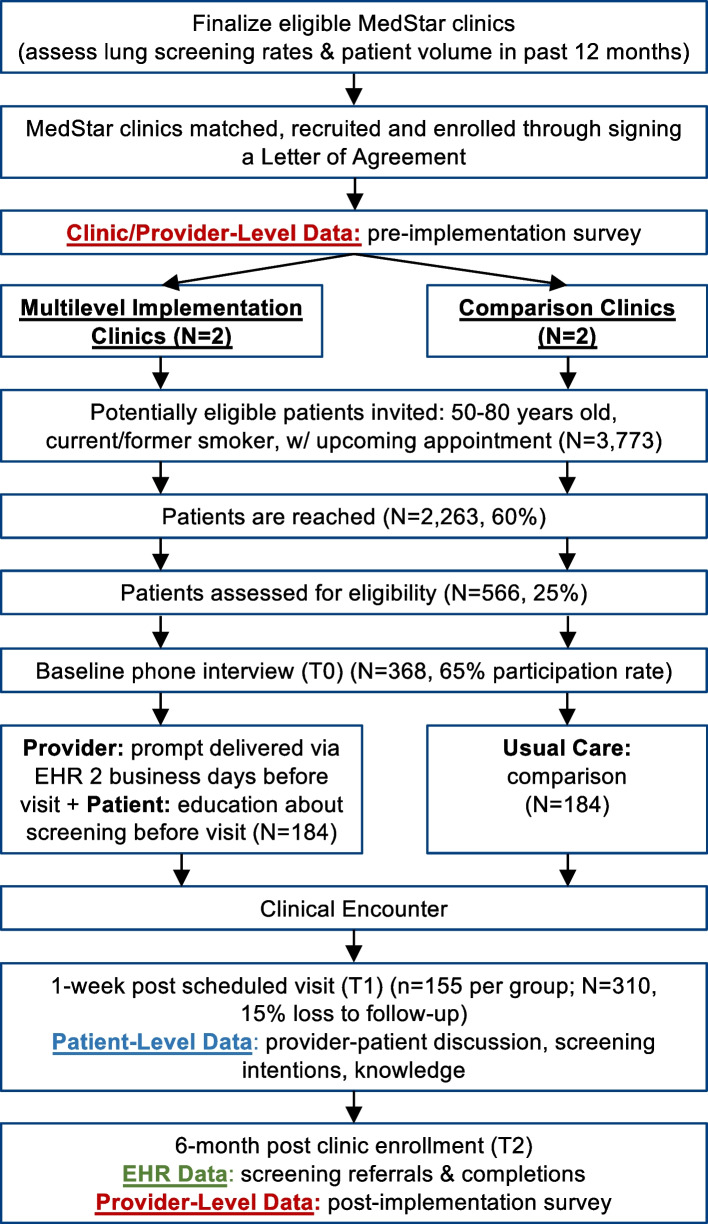
Study flow

We will conduct the quasi-experimental study in partnership with four primary care, community-based ambulatory clinics within the MedStar Health system, matched on CT scanner on site (yes/no) and minority representation (> 40% Black or African American; Fig. 2). Clinic eligibility criteria include (1) clinic providers use the Message Center within MedConnect (Oracle Cerner); (2) > 400 patients who are 50–80 years old with a documented smoking history in the last 12 months; (3) > 40% African American patient population; and (4) a site champion who will serve as the primary point of contact for this study. The provider sample includes full- and part-time primary care providers at the participating clinics.

### Patient participants

Patient eligibility criteria include (1) 50–80 years old on the upcoming appointment date; (2) currently smoking cigarettes or quit within the past 15 years; 3) a ≥ 20 pack-year smoking history; (4) non-adherent to lung screening (never screened or > 13 months since last screen); (5) English-speaking; (6) scheduled for a clinic appointment during the upcoming 3–8 weeks; (7) able and willing to provide meaningful consent; and (8) able to complete the phone-based interviews and intervention. Individuals with a prior diagnosis of lung cancer documented in the EHR will be excluded.

### Study procedures

All study procedures were reviewed and approved by the Georgetown-MedStar Institutional Review Board.

### Clinic recruitment and procedures

We have formed partnerships with primary care leadership and providers who facilitated introductions to the 4 clinical practice sites. To introduce the study, we met with practice leadership (e.g., Medical Directors, Division Chiefs) to review the study requirements. Following the introductory meetings, we received buy-in from MedStar primary care leadership (e.g., Assistant Vice President/Vice Presidents of Primary Care Services). Prior to the study start, clinics were sent a Letter of Agreement. Our team introduced the study and provided education about lung screening during an existing practice meeting. We also provided clinics on the best practices for assessing tobacco use and connecting patients with smoking cessation treatment. For the Multilevel Clinics, we notified the providers of the new clinician reminder and provided information on patient selection, when to expect the prompts and how to use them, and details about the patient component. The Comparison Clinics will identify and refer screen-eligible patients utilizing usual care as described below.

### Patient recruitment and procedures

During the recruitment period, the research assistants will access the list of potentially eligible individuals from an EHR report that our team developed to identify patients with an upcoming visit who are 50–80 years old with an ever-smoking history. Due to the limitations of the EHR smoking history data, including a large number of missing data for the pack-years field (90% on average are missing pack-years), we are conservatively estimating 25% of those reached will be eligible for the study (Fig. 2). The report utilizes EHR appointment and clinical data and will be imported into the study’s REDCap database weekly by the study team. Our team will invite patients to participate in the study through a mailed invitation letter, email, and the myMedStar patient portal, followed by phone calls (up to 3 attempts). During this call, the research staff member will describe the study, determine eligibility, obtain verbal consent, and complete the baseline assessment. We expect to enroll *n* = 184 in the Multilevel Clinics and *n* = 184 in the Comparison Clinics (total *N* = 368).

Following baseline (T0), Multilevel Clinic participants will receive the phone-based inreach and education within 1 week to 3 days prior to the visit. The comparison clinics will not receive the bundled strategies, but eligible participants will be enrolled and followed to measure the outcomes. All patient participants will receive up to $50 in total for their participation.

### Provider recruitment and procedures

In the pre-implementation phase, we will conduct semi-structured interviews to understand the contextual factors related to the feasibility and acceptability of the strategies with MedStar primary care leadership, primary care providers, RN practice managers, and medical office assistants (*n* = 25). We will conduct 30-min individual interviews which will be recorded and transcribed. During the implementation phase, the study team will send the EHR message to the primary care providers in the Multilevel Clinics 2 business days before the screening-eligible patient's scheduled visit. The messages will notify providers of the patient’s eligibility and encourage discussion of the benefits and limitations of screening. Post-implementation, we will conduct semi-structured interviews with the same participants from the pre-implementation phase. All provider participants will receive up to $100 in total for their participation.

### Multilevel implementation strategies development

Reported elsewhere [44], we completed developmental phases to finalize the content of the provider-level prompt and the patient-level inreach and education component. These phases were iterative in nature and included an online survey of *n* = 22 MedStar primary care providers, usability testing (*n* = 7), clinic shadowing to understand provider workflow, and partnering with a MedStar primary care clinic (Mitchellville, MD) for a 6-month period to optimize and refine the provider prompt. Similarly, we used a multi-step process for the patient-level component to adapt two existing, evidence-based tools: the ShouldIScreen decision aid [45] and the T.A.L.K. Back! model [46] to promote patient activation. We received feedback on the patient education component and the participant-facing materials from a Patient Advisory Board and conducted pilot testing with screen-eligible patients (*n* = 23) to refine the strategy described below.

### Patient-level inreach and education

The patient-level component includes receiving the educational booklet and a 20-min phone-based inreach session from a trained specialist approximately 1 week before the scheduled clinic visit. The information in the booklet and phone script covers the risk of developing lung cancer, along with how participants manage this risk by engaging in lung screening and smoking cessation. Additionally, it addresses self-efficacy by providing information on the lung cancer screening process. The provided resources cover possible barriers to screening (e.g., stigma from smoking, medical mistrust) and emphasize the importance of shared decision-making and discussing screening with their provider using an adapted T.A.L.K. Back! model. The session also targets patient activation by teaching good communication skills and empowering patients to feel ready to talk with their provider about lung screening.

### Provider-level reminder

**Fig. 3:**
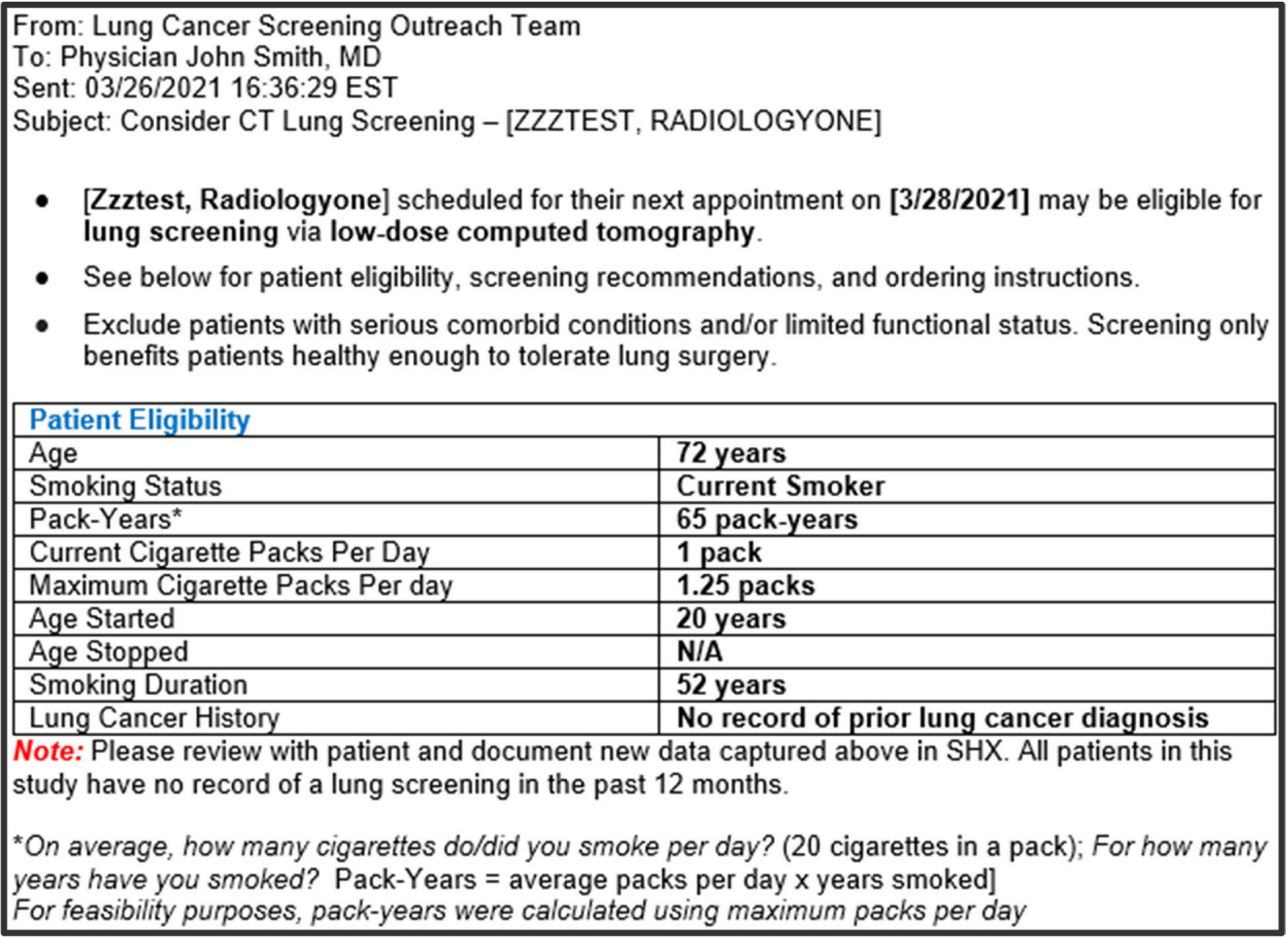
Visual of message

We will send the clinician reminder (Fig. 3) 2 days before the screening-eligible patient’s clinic visit to primary care providers from the Multilevel Clinics. The study team will build a web-hosted application to automatically pull data from an Application Programming Interface (API) to copy the baseline (T0) smoking history from the study database into a patient-specific prompt template. This prompt will be delivered through the EHR’s Message Center with a subject line, “Consider Lung Screen CT – [Patient Name]”. Based on the pilot work, the content in the body of the message includes a tabular presentation of the patient’s smoking history (e.g., age, smoking status, pack-years, age started); 2021 USPSTF recommendation; information about insurance coverage; discussion points to engage in shared decision making (including an auto-text shortcut for providers to document shared decision-making); and ordering and billing information. A set of talking points (discussion of the benefits and harms of screening, adherence to annual screening, and smoking cessation) is attached to the message. For patients currently smoking, the document includes instructions to access the order for e-referral to the tobacco quitline, the orderset of the six FDA-approved cessation medications, and prompts to encourage quitting.

### Usual care

At the time of designing this study, lung screening was not included in the list of health recommendations like other preventive services (e.g., breast cancer screening, colorectal cancer screening) in the EHR to prompt providers. Providers had to utilize the smoking history data included in the record to identify patients eligible for lung screening. However, the total pack-years field in the EHR had a large amount of missingness (90% on average are missing pack-years) across the 4 practice sites, possibly due to the field lacking automation. Usual care in MedStar primary care is expected to change as a result of ongoing related work led by our team. New forms are being released to improve the assessment of tobacco use, including automated calculation of pack-years, and to facilitate smoking cessation and lung screening referrals by displaying in the recommendations tab for eligible patients.

### Measures

We will use mixed-methods and multilevel assessments (Table 1). At the *patient-level*, participants will be assessed at baseline and 1-week post-visit to measure the primary outcomes: provider-patient discussion (“Did you have a discussion with your doctor about lung screening?”), screening intentions (“Do you plan to have a CT scan to check for lung cancer in the next 6 months?”), and knowledge using 11 true/false items from the literature [47]. We will administer the Questionnaire on the Quality of Physician–Patient Interaction scale to measure patient-reported provider-patient communication [48]. The Lung Screening Health Beliefs Scale will measure perceived risk, self-efficacy, benefits, and barriers [49]. The Consumer Health Activation Index will be administered to measure patient activation [35]. We will evaluate potential moderators including race (non-Hispanic African American vs. non-Hispanic White vs. other), smoking status (current vs. former), and health literacy (“How often do you find numbers, figures, or graphs to be useful in making a health-related decision?”) [50].

**Table 1:** Complete measures

		**T0**	**T1**	**T2**
		**Baseline**	**1 week**	**6 months**
***Patient-level variables***	*Primary outcomes*: Provider-patient discussion, lung screening intentions, screening knowledge	**X**	**X**	
	*Secondary outcomes*: Lung screening referrals, completion rates (EHR)	**X**		**X**
	*Moderators*: Race, smoking status, health literacy	**X**		
	*Intervention targets*: Physician–patient communication, perceived risk, self-efficacy, benefits/barriers, patient activation, attended visit/visit type	**X**	**X**	
	*Covariates*: Demographics, social determinants of health, tobacco behaviors, cancer screening history, psychological variables (e.g., stigma, medical mistrust)	**X**		
***Provider-level variables***	Demographics	**X**		
	Lung screening practices	**X**		**X**
	Lung screening attitudes	**X**		**X**
	Intervention acceptability, usability, intervention, effectiveness			**X**
***Implementation variables***	Reach: % of patients offered trial enrollment and the % enrolled out of the number of eligible patients who had a visit during the recruitment period	**Pre-post implementation**		
	Effectiveness: screening referrals and screening completion rates			
	Adoption: % of messages opened by the receiving provider; fidelity of the patient component			
	Implementation: feasibility, acceptability			

At the *provider-level*, we will assess provider demographics (age, race and ethnicity, years in practice, academic appointment), checklist of the current lung screening process, and attitudes toward lung screening using semi-structured interviews pre-post trial. We will use items from the validated Technology Acceptance Model to measure the usability and effectiveness of a technology-oriented strategy [51].

Secondary outcomes, screening referrals, and screening completion rates will be extracted from the EHR database at 6-month post clinic enrollment. To measure temporal trends at the *clinic-level,* lung screening orders and completion rates will also be collected 12-month pre- and post-study. Additionally, in pre-implementation, we conducted semi-structured interviews with the Medical Directors and Operations Managers for contextual purposes. We asked questions about current practice volume, standard rooming practice, workflow for reviewing and addressing health recommendations for lung cancer screening and other preventive services, and information on screening referrals and follow-up.

### Implementation outcome measures

To examine reach, we will assess the proportion of patients offered trial enrollment and the proportion enrolled out of the number of eligible patients who had a visit during the recruitment period (Fig. 1). We will examine the adoption of the provider prompt (% of messages opened by the receiving provider) and fidelity of the patient component (coding a random selection of phone sessions to assess presence/absence of behavioral targets). To understand the contextual factors related to the feasibility and acceptability of the strategies [52], in pre- and post-study, we will conduct semi-structured interviews with stakeholders across the MedStar primary care network including at the 4 study clinics. Finally, we will randomly select 30 study participants (15 Black/African American, 15 White; patients who received a LCS order and completed it and those who received a LCS order and did not complete it) to conduct in-depth interviews at 6 months to assess perceptions of the quality of the patient inreach and education, the impact of the implementation strategy, and suggestions for improvement.

### Sample size and power

All power calculations were performed for the primary outcomes (provider-patient discussion, screening intentions, and knowledge), using intention-to-treat principles, and a significance level of 0.05. We will have 83% power to detect a difference of 12% for the provider-patient screening discussion, a difference of 15% for likely screening intention, and an effect size of 0.33 for the screening knowledge score. For the moderation analyses, we will have > 80% power to detect an effect size of 0.50 with equal subgroups (e.g., Black vs White, high vs. low literacy levels).

### Statistical analysis plan

We will examine the distributions of all variables to describe the characteristics of the study population. We will conduct analyses using *t*-tests, *χ*^2^ tests, and Pearson or Spearman correlation coefficients to assess the bivariate associations between the baseline characteristics and the outcome variables. Bivariate analyses will also be conducted to assess the associations between the study arm and baseline characteristics. Owing to any significant differences detected at the 0.05 level, the variables associated with both the study arm and the outcome of interest will be entered as covariates in subsequent analyses. To test the overall difference between the study arms on the primary outcomes, we will conduct logistic regression models (for provider-patient discussion — yes/no, screening intentions — likely/unlikely) and a linear regression model (for the screening knowledge score). Logistic regression analyses will also be conducted on the secondary screening outcomes (lung screening referral — yes/no, lung screening completion — yes/no). This study will explore potential moderators (e.g., race, health literacy, smoking status). That will be done by evaluating the presence of interactions between the moderators and study arm, one at a time, after adding them to the regression models described above. In sensitivity analyses, we will evaluate the robustness of our findings by using versions of the above models with generalized estimating equations (GEE). These models will use the sites as clusters, and assume an exchangeable working correlation structure.

For the qualitative analysis, we will use a deductive thematic analysis based on a priori PRISM constructs, with additional codes added as needed. Initial codes will be generated by two coders and each of the coders will review two transcripts for each sub-population (i.e., patient and provider) to reach a consensus on the coding framework and application of codes prior to splitting up the remaining transcripts.

## Discussion

Guided by the Health Disparities Research and PRISM frameworks that take into account the multilevel determinants of cancer screening disparities and the multilevel interactions needed to understand and address these disparities, this quasi-experimental study will test the effect of provider- and patient-level implementation strategies to improve patient-provider discussion about lung screening and increase equity in lung screening rates between African American and White primary care patients. Through mixed-methods and multilevel assessments, this study is well-positioned to evaluate the impact of a provider prompt as well as a patient inreach and education component on the adoption of lung screening as an evidence-based intervention.

The planned study has three innovative aspects. First, this study will be one of the first to test the impact of multilevel implementation strategies on lung screening discussions and screening rates. We will test provider prompts that will serve as a reminder to discuss screening, and simultaneously target the patient level to address knowledge, communication, and psychological barriers to screening. It is well established that there are multiple levels of influence involved in lung screening; however, few studies have intervened beyond the individual level [24–27]. Second, the proposed study is applying the Health Disparities Research Framework to target two levels of influence, the patient-level and provider-level, that contribute to disparities in screening outcomes. This study is grounded in the disparities framework and these factors will be examined in moderation analyses. Finally, this study is testing the impact of implementation strategies aimed at the equitable adoption of lung cancer screening in diverse primary care practice settings. This study will examine the effect of these strategies on guideline-concordant provider-patient screening discussion and behaviors. The proposed strategies have the potential to increase adoption of this evidence-based intervention, improve outcomes, and mitigate racial disparities between African American and White patients who are eligible for lung screening.

This study will provide preliminary data on the effectiveness and feasibility of multilevel implementation strategies to improve lung screening utilization in an equitable manner. Future work will include a large 2 × 2 factorial, cluster randomized trial to compare the independent and overlapping contributions of the provider and patient implementation strategies. Study limitations include the quasi-experimental study design, which will limit the ability to determine causality. Second, we considered recruiting all potentially eligible patients irrespective of having an upcoming appointment, as those with a scheduled appointment may be sicker and/or more engaged in their healthcare. However, a primary endpoint is whether the provider-level prompt and the patient-level activation impact the discussion about lung screening at the clinical encounter, and so we elected to include an upcoming appointment as an eligibility criterion. It is well documented that the collection of smoking-related data (e.g., smoking duration, cigarettes per day, pack-years) in the EHR is not standardized and is often incomplete. The proposed study will use the EHR data to identify potentially eligible individuals based on age (50–80 years old) and smoking status (current and former). However, there will be a high number of screen failures as well as patients who may be eligible for lung screening but are not approached for the study due to data missingness. In other ongoing work, our research team is conducting a quality improvement project to improve how tobacco use is assessed and documented in the primary care setting across MedStar.

Following the successful completion of this study, future work will include partnering with an expanded number of primary care practices and utilizing a cluster randomized controlled trial to determine if these implementation approaches facilitate an increase in lung screening rates equitably.

## Data Availability

The datasets during and/or analyzed during the current study available from the corresponding author on reasonable request.

## Abbreviations

EHR: Electronic Health Record
USPSTF: United States Preventive Services Task Force
NLST: National Lung Screening Trial
African Americans: Black or African American individuals
Whites: White individuals
NIMHD: National Institute on Minority Health and Health Disparities
ERIC: Expert Recommendations for Implementing Change
PRISM: Practical, Robust, Implementation & Sustainability Model
RE-AIM: Reach, Effectiveness, Adoption, Implementation, Maintenance Framework
API: Application Programming Interface
GEE: Generalized Estimating Equations
